# Prospects for Advancing Tuberculosis Control Efforts through Novel Therapies

**DOI:** 10.1371/journal.pmed.0030273

**Published:** 2006-08-01

**Authors:** Joshua A Salomon, James O Lloyd-Smith, Wayne M Getz, Stephen Resch, María S Sánchez, Travis C Porco, Martien W Borgdorff

**Affiliations:** 1 Department of Population and International Health, Harvard School of Public Health, Boston, Massachusetts, United States of America; 2 Harvard Initiative for Global Health, Cambridge, Massachusetts, United States of America; 3 Department of Environmental Science, Policy, and Management, University of California Berkeley, Berkeley, California, United States of America; 4 Center for Infectious Disease Dynamics, Pennsylvania State University, University Park, Pennsylvania, United States of America; 5 California Department of Public Health Services, Tuberculosis Control Branch, Berkeley, California, United States of America; 6 KNCV Tuberculosis Foundation, The Hague, Netherlands; 7 Department of Infectious Diseases, Tropical Medicine, and AIDS, University of Amsterdam, Amsterdam, Netherlands; University of California in San Francisco, United States of America

## Abstract

**Background:**

Development of new, effective, and affordable tuberculosis (TB) therapies has been identified as a critical priority for global TB control. As new candidates emerge from the global TB drug pipeline, the potential impacts of novel, shorter regimens on TB incidence and mortality have not yet been examined.

**Methods and Findings:**

We used a mathematical model of TB to evaluate the expected benefits of shortening the duration of effective chemotherapy for active pulmonary TB. First, we considered general relationships between treatment duration and TB dynamics. Next, as a specific example, we calibrated the model to reflect the current situation in the South-East Asia region. We found that even with continued and rapid progress in scaling up the World Health Organization's DOTS strategy of directly observed, short-course chemotherapy, the benefits of reducing treatment duration would be substantial. Compared to a baseline of continuing DOTS coverage at current levels, and with currently available tools, a 2-mo regimen introduced by 2012 could prevent around 20% (range 13%–28%) of new cases and 25% (range 19%–29%) of TB deaths in South-East Asia between 2012 and 2030. If effective treatment with existing drugs expands rapidly, overall incremental benefits of shorter regimens would be lower, but would remain considerable (13% [range 8%–19%] and 19% [range 15%–23%] reductions in incidence and mortality, respectively, between 2012 and 2030). A ten-year delay in the introduction of new drugs would erase nearly three-fourths of the total expected benefits in this region through 2030.

**Conclusions:**

The introduction of new, shorter treatment regimens could dramatically accelerate the reductions in TB incidence and mortality that are expected under current regimens—with up to 2- or 3-fold increases in rates of decline if shorter regimens are accompanied by enhanced case detection. Continued progress in reducing the global TB burden will require a balanced approach to pursuing new technologies while promoting wider implementation of proven strategies.

## Abstract 2

Mathematical modeling suggests that new tuberculosis treatments that are shorter than the current 6-month standard regimen would lead to considerable reductions in incidence and mortality of TB, which remains the leading cause of global deaths.

## Editors' Summary

**Background.**

One third of the world's population is infected with *Mycobacterium tuberculosis,* the bacterium that is the main cause of tuberculosis (TB). In most people, the infection remains dormant, but every year eight million people develop active TB, usually in their lungs, and two million people die from the disease. For most of the second half of the 20th century, TB was in decline, particularly in developed countries, due to the availability of powerful antibiotics. Recently, however, global efforts to control TB have been set back by the HIV/AIDS epidemic—people with damaged immune systems are very susceptible to TB—and the emergence of antibiotic-resistant bacteria. In the 1990s, the World Health Organization (WHO) introduced DOTS as the recommended strategy for global TB control. Central to DOTS is “directly observed short-course chemotherapy.” To cure TB, several antibiotics have to be taken daily for 6 months. Patients must complete this treatment, even if they feel better sooner, to prevent relapse and the emergence of drug-resistant bacteria. The DOTS approach ensures that patients do this by having trained observers watch them swallow each dose of their medication for the entire 6-month period.

**Why Was This Study Done?**

This year, WHO and partners launched a renewed Global Plan to Stop TB, which aims to reduce, by 2015, the number of people who are sick with TB (disease prevalence) and the number of people who die each year from the disease (mortality) to half the 1990 levels. Because sick people often infect others, reducing disease prevalence will also reduce the number of new infections each year (disease incidence). The Global Plan to Stop TB includes a commitment to expand and intensify the DOTS strategy (in the year 2004, only around half of new active, infectious TB cases were detected under DOTS). The drug combinations currently used for DOTS consist of four or more different antibiotics, which have all been around and in use for many years. Recently, renewed effort has gone into the search for new TB treatments. Several candidate drugs have been identified and are now being tested, and scientists expect that some of them will be able to cure patients quicker than the current 6-month regimen. In this study, the researchers wanted to understand the potential benefits of such shorter treatments.

**What Did the Researchers Do and Find?**

The researchers developed a mathematical model that considers the acquisition, progression, diagnosis, and treatment of M. tuberculosis infection and its clinical consequences. They used the model to predict how changes in treatment duration might affect TB incidence, prevalence, and mortality. In the model, shorter treatment durations are connected to higher cure rates, as each additional month of treatment means more time to ensure that patients keep taking their medications, and more doses that might be missed. Since patients who drop out of treatment early can continue to infect other people, the model shows how a 2-month course of antibiotics will produce a quicker decline in the incidence of TB than a 6-month course by reducing these opportunities to infect others.

  The researchers then refined their model to include current conditions in South-East Asia, an area where DOTS is being scaled up and where one-third of all new TB cases occur. This “calibrated” model indicates that even if DOTS is scaled up as planned, shorter drug courses would still reduce TB incidence and deaths much quicker than a 6-month course. If, for example, a 2-month drug treatment were introduced by 2012, it might prevent 13% of the new cases and 19% of the TB deaths that would otherwise occur in South-East Asia between 2012 and 2030. These benefits might be even greater if the new drug regimen freed up resources to improve the systematic effort to detect new TB cases. On the other hand, delaying its implementation until 2022 would erase three-quarters of the predicted benefits.

**What Do These Findings Mean?**

Like all mathematical models, this one makes many assumptions that, if incorrect, will change the predictions. With this caveat, the study confirms that the planned scale-up of DOTS will greatly reduce TB incidence and mortality over the next few years. However, it also suggests that the impact of DOTS could be substantially improved by introducing shorter drug regimens. The earlier such shorter treatments become available, the greater their benefits would be. By reducing the opportunities for patients to default on their treatment and by shortening the period during which they can infect others, the researchers predict that the rate of decline in TB incidence and mortality could be up to three times higher for antibiotics that only have to be taken for 2 months compared with those that need to be taken for 6 months. But, they stress, strategies for reducing the global TB burden must strike a balance between pursuing new treatment and detection strategies and promoting wider implementation of proven strategies. And while there is hope that new shorter treatments will prove effective over the next few years, this will only become clear as candidates are rigorously tested.

**Additional Information.**

Please access these Web sites via the online version of this summary at http://dx.doi.org/10.1371/journal.pmed.0030273.

• US National Institute of Allergy and Infectious Diseases patient fact sheet on TB

• US Centers for Disease Control and Prevention information for patients and professionals on TB

• MedlinePlus encyclopedia entry on TB

• NHS Direct Online patient information on TB from the UK National Health Service

• World Health Organization information on global TB control

• Global Alliance for TB Drug Development information on current initiatives to develop new TB drugs

• Wikipedia page on TB treatment (note that Wikipedia is a free online encyclopedia that anyone can edit)

## Introduction

Tuberculosis (TB) persists as a leading global cause of death. In 2003, nearly 9 million new cases of active pulmonary TB and 2 million deaths from TB were reported [1]. After major and sustained progress in reducing the burden of TB—marked by declines for over a century in some industrialized countries [2–4]—global TB control efforts have faced new challenges due to the HIV epidemic and the rise of drug resistance in several settings [5,6].

Following the Millennium Declaration, endorsed by consensus of the members of the United Nations [7], targets relating to global TB control have been guided by the Millennium Development Goals—specifically, the aim of having “halted by 2015 and begun to reverse the incidence of malaria and other major diseases” [8]. Extending this objective to include other indicators of progress against TB beyond reductions in incidence, the Stop TB partnership further resolved to halve TB prevalence and death rates between 1990 and 2015 [9].

The cornerstone of global TB control efforts led by the World Health Organization (WHO) is the DOTS strategy, which relies on short-course chemotherapy for individuals with active pulmonary TB, with drugs administered under the direct observation of health workers. Case detection in DOTS is based on identifying individuals who present to health services with persistent cough, and screening them by examination of sputum smears. Cure rates above 80% have been achieved using DOTS [1], but concerns have been raised about the scope and pace of DOTS expansion and potential limits to the reach of passive case detection under DOTS [10].

Current standardized regimens for treating TB require four or more drugs administered for at least six months [11]. No new first-line TB drugs have been developed in over 30 years. Shorter regimens including novel drugs have the potential to advance TB control efforts by speeding treatment completion—thereby limiting opportunities for default and the potential emergence of drug resistance—and reducing relapse rates. Shorter regimens may also facilitate expanded case-detection efforts by freeing scarce resources and lowering patient-related barriers to initiating therapy. These effects would be expected to translate directly into better outcomes for those receiving treatment, and indirectly into lower TB incidence and mortality due to reduced transmission.

As new drug candidates emerge from the global TB drug pipeline [12], the potential exists to modify or replace existing regimens with faster-acting drugs that could reduce treatment time, work concomitantly with HIV therapies, and overcome multidrug resistance. An array of candidates has been identified, including both new chemical entities and compounds from existing drug classes. Examples of the former include the nitroimidazole PA-824 [13,14], which began Phase I trials in 2005, and the diarylquinoline R207910, which was reported to yield complete culture conversion within two months in an animal model when substituted into existing drug combinations [15]. Examples of the latter include moxifloxacin and gatifloxacin [16,17], already licensed for other indications.

In order to examine the expected benefits of shortened drug regimens, we developed a mathematical model of TB to gain general insights into the impact of treatment duration on TB dynamics, and to simulate various possible trajectories of the epidemic under continued application of existing tools, or addition of new technologies that may be developed in the future. To provide a specific regional example, we calibrated the model to epidemiologic data for WHO's South-East Asia region, which includes five of the 22 highest-burden countries and accounted for approximately one-third of the estimated TB cases and deaths in 2003 [1].

## Methods

### Basic Model

We developed a dynamic compartmental model of TB (Figure S1), expanding on previous models that have been used to examine the natural transmission dynamics of TB and to evaluate alternative TB control strategies [18–21]. In particular, we adapted the model presented by Dye et al. [18] and preserved a number of key assumptions from that model in order to build on their previous analysis of the potential impact of DOTS with currently available regimens. Mathematical details of the model and values for key parameters relating to the transmission and natural history of TB are presented in Protocol S1.

The model includes acquisition of infection, progression to active pulmonary TB, diagnosis, and treatment. New infections may lead to primary progression or to latent infection, which is subject to endogenous reactivation or exogenous reinfection. A previous infection is assumed to confer partial immunity against reinfection.

Active pulmonary TB is categorized as smear-positive or smear-negative, which are associated with different degrees of infectiousness, different natural history parameters (governing spontaneous cure and TB fatality), and different treatment outcomes. Untreated active cases may spontaneously resolve and return to latent infection. A fraction of new active cases are eligible for detection at a rate that reflects the delay from disease onset to diagnosis. Both the proportion eligible for detection and the detection rate may vary between smear-negative and smear-positive cases.

We elaborated on previous models by explicitly tracking the duration of treatment in months, in order to examine the impact of shorter drug regimens. Patients who complete treatment successfully are considered cured but remain partially susceptible to reinfection. Those who discontinue treatment early (default) or fail upon completion of treatment face the same mortality risks as those without active disease, but are partially infectious and subject to relapse to active disease. Although relapsing cases may be detected and treated again, we did not explicitly track treatment history nor explicitly model distinct retreatment regimens.

### Treatment Duration and TB Dynamics

To gain general insights into the relationships between treatment duration and TB dynamics, we first modeled the introduction of treatment into a stable equilibrium, following the example of Dye and colleagues [18]. We undertook a range of different simulations, varying key parameters relating to treatment programs, including duration, default rates, and the probability of remaining infectious after treatment failure. Considering treatment regimens of 2-, 4-, and 6-mo durations, we estimated the fatality probability for a treated case; the average cumulative duration of infectiousness for a new case of smear-positive TB; the number of secondary infectious cases generated by an average infectious case; and changes in annual incidence rates after introduction of treatment into steady-state conditions. Details on the calculations supporting the estimation of each of these quantities are reported in Protocol S1.

### Expanded Model and Calibration to South-East Asia

To consider key features of TB epidemics in specific current settings, we expanded the basic model to allow for heterogeneous treatment, development of drug resistance, and interactions between TB and HIV (see Protocol S1).

The model accommodates two different varieties of treatment, for example representing DOTS and other programs, with allowance for different trends in coverage over time and different treatment outcomes.

We allowed for development of drug resistance through poor treatment as well as transmission of resistant strains. Although various profiles of drug resistance appear in different settings [6], for parsimony we distinguished only cases that are multidrug resistant—defined by resistance to the two most potent first-line anti-TB agents (isoniazid and rifampicin). We also incorporate a simplified model of HIV-1, distinguishing between those who are infected and those who are not. Incidence rates of HIV infection over time are taken from WHO/UNAIDS estimates, with a three-year delay to capture the decline in immune function relevant to TB natural history (see Protocol S1). These additional details are included in order to allow more realistic modeling of the dynamics of real-world TB epidemics, and are reflected in the results we present here; however, specific comment on features of shorter drug regimens in relation to multidrug resistance or HIV status is beyond the scope of this report.

Parameters in the model were calibrated to provide a close fit to estimated TB incidence and mortality in WHO's South-East Asia region, which includes India, Indonesia, Bangladesh, Myanmar, and Thailand among the 22 highest-burden countries. Calibration was undertaken in three stages. First, we simulated a natural history TB epidemic until a stable equilibrium was attained. This equilibrium was the same as that used in the general analyses of treatment duration and disease dynamics described above. Second, we introduced treatment representing the beginning of national TB control programs in countries such as India starting in the 1960s [22]. Third, we modeled the initiation of DOTS in the 1990s based on reported program data on coverage and treatment outcomes. Epidemiologic indicators for the South-East Asia region were obtained from routine surveillance estimates by WHO [1]. Treatment patterns and results were based on reports from national TB control programs (see Figure S2 and Protocol S1) [1].

### Scenario Analyses

Different scenarios were defined to include alternative assumptions about case detection coverage, cure rates, and the introduction of new, shorter drug regimens. Alternative scenarios relating to continued progress in scaling up DOTS were modeled with a starting date of 2002, and introduction of new drug regimens was modeled with a starting date of 2012.

### Baseline Scenarios

We considered two different scenarios relating to continued application of DOTS with current regimens. The scenarios, reflecting different case-detection probabilities, are defined in the model by varying the proportion of new cases eligible for detection under DOTS and non-DOTS programs (see Figure S2). For ease of practical implementation this parameterization of case detection in the model departs slightly from the case detection statistics reported by WHO (number of new cases detected divided by estimated incident cases in a given year) but will approximate the reported case detection ratio when trends in incidence and coverage are relatively steady.

In the stable-DOTS scenario, scale-up of DOTS follows reported trends through 2003 but stabilizes at the 2003 level (with around 42% case detection coverage for new smear-positive pulmonary cases). In the DOTS-target scenario, we assumed that the WHO target of 70% case detection is attained by 2009. Following Dye et al. [18], we assumed in both the stable-DOTS and DOTS-target scenarios that detection of smear-negative TB cases occurs in constant proportion (60%) to detection of smear-positives.

### New Drug Scenarios

To analyze the possible impact of new, shorter drug regimens, we considered the benefits of a 2-mo regimen, including in the first instance only the reductions in the cumulative probability of default that would be attributable to earlier treatment completion. We assumed that with either current regimens or new, shorter regimens, monthly default rates are 1.5% in DOTS treatment programs and 7.5% in non-DOTS treatment programs, and that failure probabilities for those completing a full treatment regimen are 3% in DOTS programs and 6% in non-DOTS programs. Combined with mortality risks, these assumptions translate into overall cure probabilities for current regimens of 85% in DOTS treatment programs and 50% in non-DOTS programs, consistent with assumptions reported for this region by Dye et al. [18]. For shorter regimens, the same monthly default assumptions and failure probabilities applied over two months yield cure probabilities of 93% and 80% in DOTS and non-DOTS programs, respectively.

As an alternative set of scenarios relating to introduction of new drug regimens, we further considered the potential for shorter drug regimens to be accompanied by broader case detection through the freeing of scarce financial and human resources and the lowering of barriers to initiation of therapy. These additional benefits are not certain to arise, but their likelihood would be enhanced by development of new diagnostic technologies. Specifically, we examined the potential incremental benefits that would be gained if the proportion of undetected cases were reduced by half after introduction of new drugs.

### Sensitivity and Uncertainty Analysis

Uncertain model parameters were varied in sensitivity analyses, and uncertainty analyses were conducted in order to provide ranges around model outputs. We used a multiple simulation approach in which parameter values were sampled from defined ranges and the model was recalculated under each set of sampled parameter values. Sensitivity analysis was used to identify those parameters that had the greatest impact on the outcomes of interest. Ranges around estimates are reported based on the 2.5th and 97.5th percentiles across the set of simulations (see Protocol S1).

## Results

### General Implications of Treatment Duration

We first evaluated the general relationship between treatment duration and TB dynamics by simulating the introduction of treatment into a steady-state equilibrium, following Dye and colleagues [18]. To identify an upper bound on the benefits of treatment, we modeled complete coverage in the population and assumed (as in [18]) that scale-up of treatment occurs in just one year. In this preliminary analysis, we considered only treatment of smear-positive pulmonary cases.

**Figure 1:**
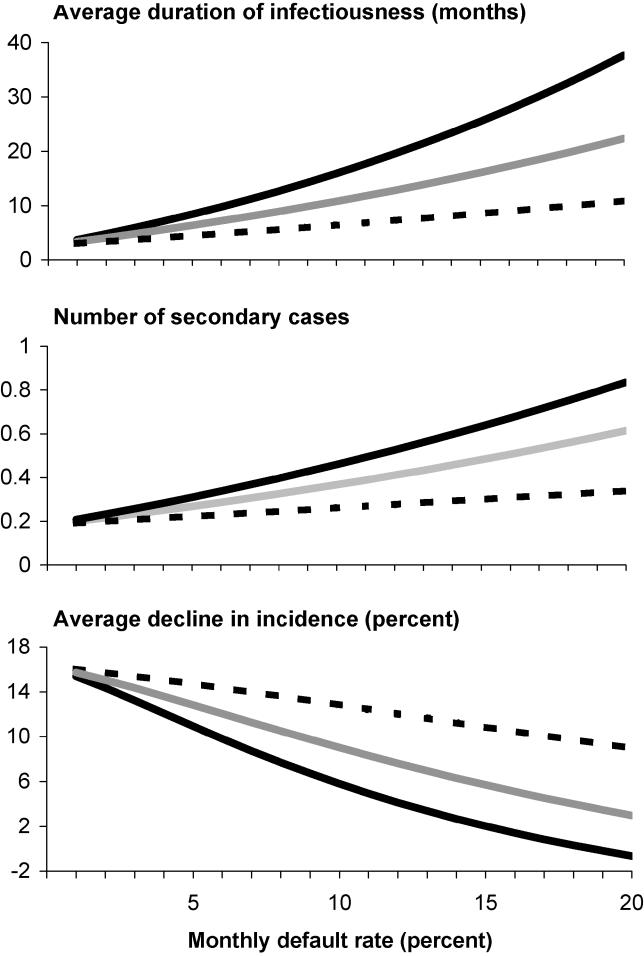
Impact of Treatment Duration on TB Dynamics The horizontal axis in each plot shows monthly default rates, and the three lines represent treatment durations of 6 mo (solid black line), 4 mo (solid grey line), and 2 mo (dashed black line). The three panels show model estimates for: (top graph) the average cumulative duration of infectiousness for a smear-positive case; (middle graph) the effective reproductive number measured 20 years after treatment is introduced; and (bottom graph) the annual rate of decline in the incidence of active TB, averaged over the 20 years following treatment introduction.

The benefits of shortening regimens are best understood by examining both direct impacts on improved treatment outcomes due to accelerated treatment completion, and indirect benefits of reducing transmission due to shortened average duration of infectiousness for treated cases. Figure 1 compares the implications of treatment regimens with different durations and default rates, with respect to three different indicators: (1) the average cumulative duration of infectiousness for a smear-positive case, accounting for the potential for multiple cycles of active disease, treatment, and relapse; (2) the expected number of secondary infectious cases generated by the average infectious case (effective reproductive number), measured at 20 years after the introduction of treatment; and (3) the mean annual rate of decline in the incidence of active TB, computed for each year relative to the previous year and then averaged over the 20 years following treatment introduction.

The benefits of shortening the duration of therapy are greatest when default rates are highest. With a monthly default rate of 15%, for example, the expected cure rates for regimens of 6, 4, and 2 mo would be approximately 40%, 50%, and 70%, respectively. Under these circumstances, average declines in incidence would be three times higher for a 4-mo regimen compared to a 6-mo regimen (adding around 4% to the average annual rate of decline in absolute terms), and six times higher for a 2-mo versus a 6-mo regimen (speeding declines by around 10% in absolute terms). However, even at relatively low default rates, shorter regimens are expected to enhance the epidemiologic impact of therapy. For example, with a default rate of 3% per month—implying an 80% cure probability for a 6-mo regimen—the average annual rate of decline in incidence would be 8% greater for a 4-mo regimen, or 14% greater for a 2-mo regimen, compared to a 6-mo regimen (1% or 2% increases, respectively, in absolute terms).

A critical reason that treatment default leads to poorer epidemiologic outcomes is that incomplete treatment may reduce mortality rates without eliminating infectiousness. In our base scenario analyses (Figure 1), we assumed that 50% of people who default from treatment would remain infectious, but this assumption remains uncertain. We therefore examined alternative assumptions in sensitivity analyses (Figure S3). If only 25% of people who default from treatment remain infectious, the benefits of shortening regimens from 6 mo to 2 mo would be marginally lower; for example, at a monthly default rate of 15%, the absolute difference in the pace of incidence decline under the shorter regimens would be around 7% rather than 10%. If 75% of defaulters remain infectious, on the other hand, benefits of shorter regimens would be even greater—with declines around 12% faster in absolute terms for a 2-mo regimen, assuming a monthly default rate of 15%.

**Figure 2:**
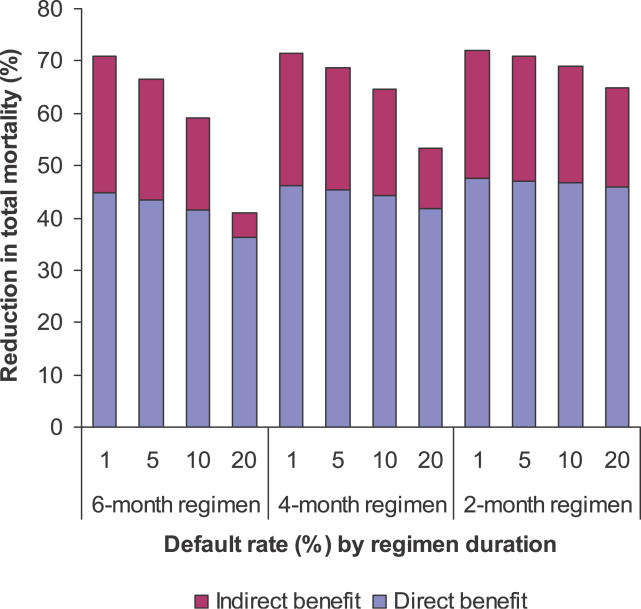
Direct and Indirect Reductions in Mortality in Relation to Treatment Duration and Monthly Default Rates Each bar shows the total reduction in deaths over a 20-year period following treatment introduction, expressed as a percentage of the deaths expected in a steady-state, no treatment counterfactual. The blue area in each bar indicates the reduction that is attributable directly to benefits in treated patients.

The total impact of treatment on mortality includes both direct benefits to those persons who are treated and further reductions in overall mortality due indirectly to reduced incidence (Figure 2). In general, the proportion of the total mortality reduction that is attributable to direct benefits of treatment rises as default rates rise (and hence cure rates fall), since mortality benefits attenuate less than transmission benefits under incomplete treatment. Mortality reductions under shorter regimens are less sensitive to the default rate, as expected, because opportunities for default are reduced as treatment duration is shortened.

### Scenario Analyses in South-East Asia

**Figure 3:**
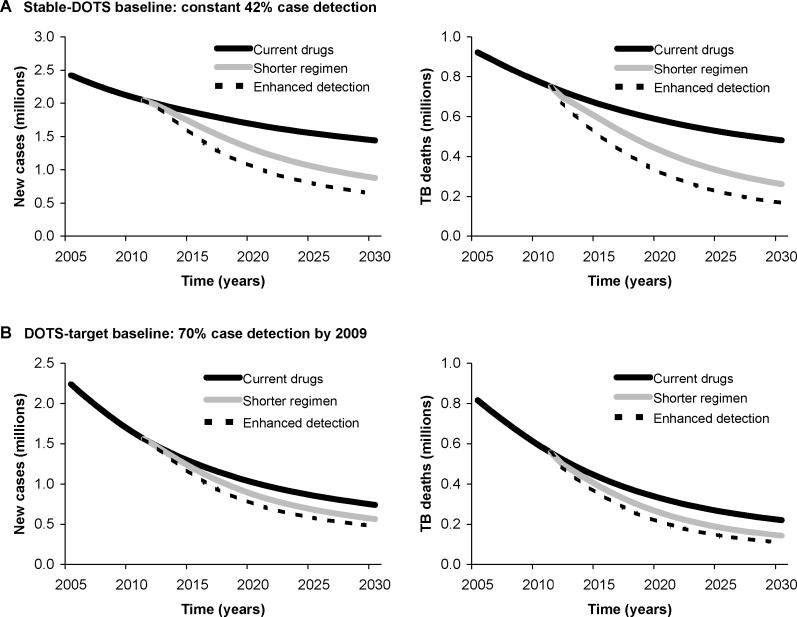
Projected Trends in New TB Cases and Deaths in the WHO South-East Asia Region, 2005–2030 (A) Benefits of new drugs and enhanced detection are projected in comparison to the stable-DOTS baseline (constant 42% case detection). (B) The same benefits as in (A) are projected in comparison to the DOTS-target baseline (70% case detection by 2009).

To examine the potential impact of new drugs in a particular epidemiologic setting, we calibrated the model to data for the South-East Asia region. Two different baseline scenarios represented alternative trajectories in the scale-up of DOTS in this region (see Figure S2). If DOTS coverage continues at current levels (stable-DOTS baseline), the numbers of new TB cases in the South-East Asia region are expected to decline by an average of 2.1% (range 0.0%–3.2%) per year over the period 2005 to 2030, with similar declines (2.6% per year, range 0.0%–3.8%) expected for deaths. If the WHO target of detecting 70% of new smear-positive TB cases is reached by 2009 (DOTS-target baseline), cases and deaths would decline by an average of 4.3% (range 1.3%–6.1%) and 5.1% (range 1.2%–6.8%) per year, respectively, over this period (Figure 3). Under these two alternative baseline scenarios the total numbers of cases and deaths over this period would be, respectively, around 50 million and 20 million in the stable-DOTS scenario, or around 30 million and 10 million in the DOTS-target scenario.

The potential impact of new drugs was examined in terms of reductions in TB incidence and mortality through 2030, including intermediate outcomes by the year 2015, of relevance to the Millennium Development Goals. We compared a variety of different measures across scenarios to evaluate the benefits of shorter regimens: incidence and mortality rates in the years 2015 and 2030; annual rates of decline in incidence and mortality (computed in each year and then averaged over the period 2012 to 2030); and cumulative numbers of new cases and deaths between 2012 and 2030.

With the availability of a new 2-mo regimen starting in 2012, accounting only for the benefits associated with earlier treatment completion, annual incidence would be 9% (range 5%–11%) lower in 2015 and 39% (range 22%–51%) lower in 2030 than in the baseline stable-DOTS scenario. Annual mortality reductions would be similar (11%, range 7%–13%; and 46%, range 32%–54%, in 2015 and 2030, respectively). Introduction of a shorter regimen would produce more than a 2-fold increase in the rates of decline in incidence and mortality compared to the stable-DOTS baseline (Figure 3). Overall, a new drug would lower the total number of new TB cases and deaths in this region between 2012 and 2030 by more than one-fifth (Table 1).

**Table 1:**
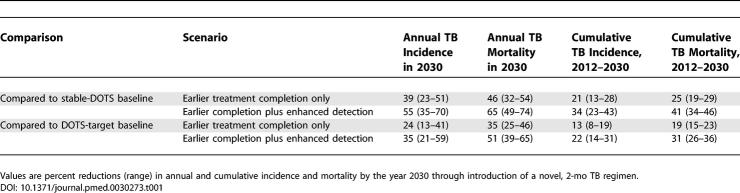
Reductions in TB Incidence and Mortality

If WHO case detection targets for DOTS are attained by 2009, introduction of a 2-mo regimen would reduce annual incidence by 6% (range 3%–8%) by 2015 and 24% (range 13%–41%) by 2030. Annual mortality rates would be 10% (range 7%–12%) lower in 2015 and 35% (range 25%–46%) lower in 2030. The rates of decline in incidence would be approximately one-third greater with new drugs than in the DOTS-target baseline, and rates of decline in mortality would be 45% greater with shorter regimens. Overall, the total reductions in new cases and deaths through 2030 would be approximately 13% (range 8%–19%) and 19% (range 15%–23%), respectively, of the 2012–2030 totals (Table 1).

We also considered the possibility that availability of shorter regimens, perhaps in combination with new diagnostic technologies, could enhance case detection efforts by reducing resource demands per treated patient, simplifying infrastructure, monitoring, and administrative requirements, and lowering patient-related barriers to initiating therapy. If new drugs and diagnostics could reduce the number of undetected cases by half, in addition to providing the benefits of earlier treatment completion as described above, the reductions in annual incidence and mortality by 2030 would be around 40%–50% percent greater; cumulative effects over the period 2012–2030 would be around 60%–70% greater (Table 1). The total effect of introducing shorter regimens, including potential enhancements in case detection, would be to accelerate the declines in incidence and mortality by a factor of around three compared to the stable-DOTS baseline, or by around 50% and 70%, respectively, compared to the DOTS-target baseline.

**Figure 4:**
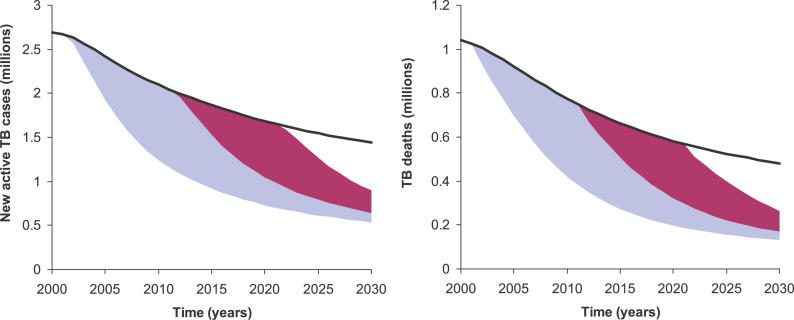
Impact of Alternative Timing for the Introduction of Shorter Drug Regimens The left graph shows projected new TB cases in the WHO South-East Asia region, while the right graph shows projected deaths. The blue area in each graph represents the additional cases and deaths that would have been averted if a new drug had been available in 2002, compared to availability in 2012. The red area shows the additional cases and deaths that can be averted if the new regimen becomes available in 2012 compared to 2022.

Thus far—considering the potential epidemiologic benefits of a 2-mo regimen available by the year 2012—we find that the total benefits through the year 2030 could be as high as 11 million cases and 5 million TB deaths averted in a scenario in which new regimens are accompanied by enhanced case detection, compared to the stable-DOTS baseline. We may also examine a retrospective counterfactual involving earlier availability of a shorter-regimen drug. If a new drug had been available starting in the year 2002, the total benefit by 2030 would be approximately double (Figure 4). On the other hand, if we consider an alternative future scenario in which a 2-mo regimen becomes available only in 2022, this delay would erase nearly three-fourths of the benefits expected from a 2012 debut (Figure 4).

## Discussion

Our analysis of the potential benefits of introducing a new, shorter drug regimen for treating active TB indicates that the impact of DOTS could be enhanced substantially with the availability of new tools. By reducing opportunities for default and failure, shorter regimens could lower annual incidence and mortality by around 40% by the year 2030; the possibility that simplified regimens combined with new diagnostics could facilitate broader case detection in DOTS programs would magnify these benefits significantly. We recognize, however, that introduction of shorter regimens will not automatically be accompanied by expanded case detection—and that various elements in a global TB control strategy must compete for scarce resources—so we emphasize that these results are simply suggestive of additional benefits that might be realized through a wider reach of case detection.

It is important to note that we have focused in this study on the example of South-East Asia, where current cure rates in DOTS programs are high, and where DOTS scale-up is proceeding relatively quickly. The TB control target arising from the Millennium Development Goals is likely to be met in this region even without the introduction of new drugs. The potential benefits of shorter drug regimens described in this report may therefore be considerably lower than those in other regions where case detection in DOTS remains minimal, or where cure rates are lower. Particularly in regions where the pace of uptake of DOTS has been slow, the potential for shorter drug regimens to lower barriers to DOTS expansion could produce even greater benefits than those reported in our analysis.

Inevitably, a model requires a series of simplifying assumptions that must be recognized. In particular, we have modeled drug resistance and interactions with HIV in a parsimonious fashion. The effect of a shorter regimen with one or more new drugs may be particularly important in settings where multidrug-resistant TB is highly prevalent, and more subtle benefits may be realized in relation to resistance patterns other than multidrug-resistant TB. However, we have not accounted for the possible development of resistance to the new drugs. With regard to HIV, the relative impact of new drugs in areas of high HIV prevalence remains uncertain. More comprehensive conclusions about the range of potential benefits of new drug regimens in other regions await ongoing analyses using extensions of the model described here.

Although we have indicated ranges around results that reflect the imprecision in modeled outcomes that follows from uncertain input parameters, these ranges do not capture all sources of uncertainty. Certain elements of the scenarios defined here, such as the potential impact of new drugs on case-detection coverage, are speculative, so results should be interpreted as “what-if” assessments rather than as firm predictions.

Nevertheless, several conclusions emerge from our analyses as important inputs into ongoing policy discussions regarding global TB control. We confirmed that large potential gains are expected if scale-up of DOTS proceeds rapidly. There is likely to remain a substantial burden due to TB, however, even under scenarios involving successful attainment of coverage targets for DOTS. Incorporation of new technologies within the DOTS strategy could dramatically accelerate the pace of declines in TB incidence and mortality expected using current regimens—with up to 2- or 3-fold increases in rates of decline if shorter regimens are accompanied by enhanced case detection. Long-term strategies for reducing the burden of disease from TB require a balanced approach to pursuing new technologies while promoting wider implementation of proven strategies.

## Supporting Information

Figure S1 Schematic of the Model Notes: (1) Mortality rates are not shown. (2) Treated cases default and move to treatment failure at rate *δ*_D_ or *δ*_N_ (specific to DOTS or non-DOTS). A fraction (*ν*_D_ or *ν*_N_) of cases completing the final month of treatment also fail. (3) A fraction (1 − *ν*_D_ or 1 − *ν*_N_) of cases completing the final month of treatment are fully recovered. (4) Only detectable cases are eligible to move from untreated to treated.
(21 KB PDF)

Figure S2 Case Detection Assumptions in Two Baseline Scenarios Stable-DOTS scenario (left graph) assumes that detection of new smear-positive cases in DOTS areas remains stable at 42% starting in 2003. DOTS-target scenario (right graph) assumes that DOTS coverage reaches 70% by 2009.
(14 KB PDF)

Figure S3 Treatment Duration and TB Dynamics: Sensitivity Analysis Graphs show the sensitivity of results in Figure 1 to the level of infectiousness after incomplete treatment. The series of graphs on the left assume that 25%, and the series on the right assume that 75%, of defaulters remain infectious. In each series, the three graphs show model estimates for: (top) the average cumulative duration of infectiousness for a smear-positive case; (middle) the effective reproductive number measured 20 years after treatment is introduced; and (bottom) the annual rate of decline in the incidence of active TB, averaged over the 20 years following treatment introduction. The horizontal axis in each plot shows monthly default rates, and the three lines represent treatment durations of 6 mo (solid black line), 4 mo (solid grey line), and 2 mo (dashed black line).
(13 KB PDF)

Protocol S1 Technical Appendix (209 KB PDF)

## Abbreviations

TB: tuberculosis
WHO: World Health Organization
