# Supplementary material for: Prospects for Advancing Tuberculosis Control Efforts through Novel Therapies
Source: PLoS Med. 2006 Aug 1;3(8):e273. doi: 10.1371/journal.pmed.0030273 (PMC1523376; doi:10.1371/journal.pmed.0030273)
Supplement: Supplementary file 1 [file pmed.0030273.sd001.pdf]

Prospects for advancing tuberculosis control efforts through novel therapies

Joshua A. Salomon, James O. Lloyd-Smith, Wayne M. Getz, Stephen Resch, María S. Sánchez, Travis C. Porco, Martien W. Borgdorff

PROTOCOL S1: Technical Appendix

Here we provide technical details of our dynamic model of tuberculosis (TB) natural history, epidemiology, and treatment. The model builds on prior modelling work on tuberculosis control and key recent studies [1–4]. We first outline a core model of TB, followed by (i) a description of the simplified version of the core model used in our general analysis of the effects of treatment duration on TB dynamics; and (ii) simple extensions to the core model that account for multi-drug resistant (MDR) TB and a simultaneous HIV-1 epidemic. We also describe methods and results for sensitivity and uncertainty analyses in order to identify parameters with the greatest impact on outcomes of interest and to estimate ranges around key model outputs.

Description of core TB model

The population is structured into classes describing different states of TB disease and treatment (Table S1). Transitions between classes are determined by the parameters described in Table S2. Figure S1 presents a simplified schematic of the model. The model is deterministic and operates in discrete timesteps, but within each timestep all processes act as constant, simultaneous, per capita rates (using a competing exponential rates formulation, described below).

All individuals are subject to natural mortality at constant per capita rate μ. Individuals who have never been infected with TB occupy the susceptible class (S), which is replenished with new recruits based on a specified population fertility rate. Susceptible individuals are infected at rate λ, the force of infection, which is calculated at the beginning of each timestep as described below. A fraction p_S of newly-infected individuals enter the latent infection/slow-progressor class (IS), while the remaining $(1–p_S)$ enter the fast-progressor class (IF). Individuals in IS are subject to reinfection at rate $b\lambda$, where b is a factor less than one reflecting reduced susceptibility. Of these reinfections, only a fraction $(1–p_S)$ move to the IF class, so the net flow from IS to IF is $b\lambda(1–p_S)$. Individuals in the IS and IF pools progress to active TB at rates ϕ_S and ϕ_F, respectively, where $\phi_S<\phi_F$.

Active TB cases are divided into four categories, depending on whether they are smear-positive or smear-negative, and whether they are in a detectable or non-detectable pool of the population (where detectability may be a function of geographical location, local health services, and case-finding effort, as well as health-seeking behaviour and intensity of symptoms). New cases are smear-positive with probability s_P or smear-negative with probability $(1–s_P)$. A fraction d_P of smear-positive cases are detectable, while for smear-negative cases the fraction is d_N. Smear-negative cases convert to smear-positive at constant rate σ, with no change in detectability. Smear-positive and smear-negative individuals are subject to additional TB-related mortality at per capita rates μ_P and μ_N, and recover spontaneously to the slow-progressor latent class (IS) at rate π. Individuals in the fully-recovered class can be reinfected at rate $b\lambda$, i.e. with the same reduced susceptibility as other previously infected persons.

Individuals in the detectable smear-positive (APd) class are identified and brought onto treatment at rate θ. Of individuals enter-

TABLE S1. State variables for TB.
The distribution of the population across different TB states is presented as a 42-element vector.

State	Abbrev.	Definition
1	S	Susceptible
2	IS	TB infection, slow progressor (latent)
3	IF	TB infection, fast progressor
4	AP	Smear-positive active TB, not detectable
5	AN	Smear-negative active TB, not detectable
6	R	Recovered
7	APd	Smear-positive active TB, detectable
8	ANd	Smear-negative active TB, detectable
9–16	TPD	Smear-positive TB case undergoing DOTS treatment, months 1-8
17–24	TPN	Smear-positive TB case undergoing non-DOTS treatment, months 1-8
25	TPF	Treatment failure, smear-positive case
26–33	TND	Smear-negative TB case undergoing DOTS treatment, months 1-8
34–41	TNN	Smear-negative TB case undergoing non-DOTS treatment, months 1-8
42	TNF	Treatment failure, smear-negative case

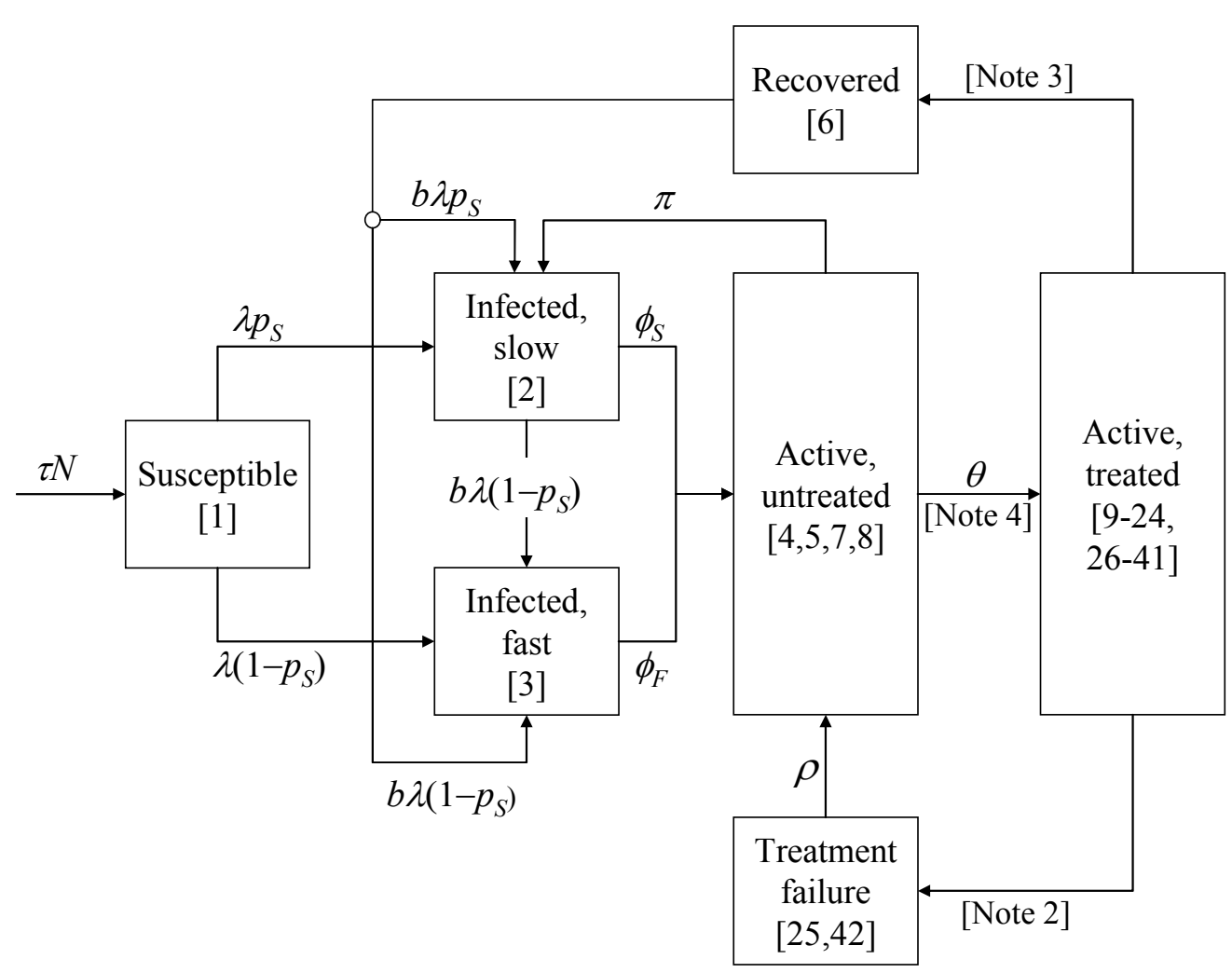

FIGURE S1. Schematic of the model.
Notes: (1) Mortality rates not shown. (2) Treated cases default and move to treatment failure at rate δ_D or δ_N (specific to DOTS or non-DOTS). A fraction (ν_D or ν_N) of cases completing the final month of treatment also fail. (3) A fraction ($1– \nu_D$ or $1– \nu_N$) of cases completing the final month of treatment are fully recovered. (4) Only detectable cases are eligible to move from untreated to treated.

ing treatment, a fraction t_P enter DOTS programs while the remainder enter non-DOTS programs. Individuals in DOTS programs are tracked through up to six months of treatment (TPD [1]–TPD[6]), and are subject to additional mortality at rate μ_{PD} throughout. Individuals default (cease treatment) during month m of treatment at rate δ_D, whereupon they move to a failed-treatment state of partially suppressed disease (TPF). Individuals who complete treatment go to treatment failure with probability ν_D or recover fully with probability ($1-\nu_D$). Individuals in the failed-treatment class are partially infectious (compared to active cases) but experience no TB-related mortality, and relapse to active TB at rate ρ. Relapsers can enter the detectable pool (APd) with probability d_P, or alternatively go to AP reflecting the possibility of losing contact with the health system.

Individuals undergoing treatment in non-DOTS programs are tracked through up to eight months of treatment (TPN[1]–TPN [8]) and are subject to the same processes as those in DOTS but with different parameters μ_{PN}, δ_N, ν_N, etc. Defaulters and treatment completers whose treatment fails enter the same partially-suppressed disease class (TPF) as do those failing from DOTS; treatment success leads to the fully-recovered class (R).

Treatment of smear-negative individuals runs in a manner precisely analogous to smear-positive cases, with DOTS (TND[1]–TND[6]) and non-DOTS (TNN[1]–TNN[8]) programs and a partially-suppressed failed-treatment class (TNF). All processes are parallel to those described above, with some parameters shared between smear-positives and smear-negatives (those subscripted only by D and N indicating DOTS and non-DOTS) and others specific to smear-negatives (subscripted ND for DOTS and NN for non-DOTS).

The force of infection is calculated at each timestep from the current number of individuals with active disease. The population is assumed to mix randomly with density-independent contact rates, so transmission is modelled as frequency-dependent. All individuals with untreated smear-positive TB transmit at rate β_P, while those with untreated smear-negative TB transmit at rate β_N. Smear-positive cases that fail treatment (TPF) have partially-suppressed disease and transmit at a fraction of the rate of active cases ($\beta_{PF}=0.5\times\beta_P$). Individuals undergoing DOTS treatment, and smear-negatives who fail treatment are assumed to be non-infectious ($\beta_{PD}=\beta_{ND}=\beta_{NF}=0$), while those in non-DOTS programs (assumed to have lower drug adherence, on average) transmit at 25% the rate of untreated cases ($\beta_{PN}=0.25\times\beta_P$ and $\beta_{NN}=0.25\times\beta_N$).

Model formulation

The model is updated in one-month timesteps. We have used this discrete formulation because it is better suited to modeling precise durations of treatment than a continuous formulation using ordinary differential equations. When a group of individuals is subject to multiple processes simultaneously (e.g. individuals in the latent fast-progressor (IF) class can either die or break down to active disease), their outcome in any timestep is determined using a competing rates formulation, as described below. This approach has the advantage that an artificial order of processes does not have to be defined because all processes act simultaneously, but it does introduce the implicit assumption that each individual can only undergo one state transition per timestep.

Competing rates example

Consider a pool of individuals C, subject to two competing processes: they go to class A at per capita rate α, and to class G at per capita rate γ. The differential equation describing class C is thus:

$$\frac{dC}{dt} = -(\alpha+\gamma)C$$

This simple equation can be solved exactly, yielding:

$$C(t+\Delta t) = C(t)\exp\left[-(\alpha+\gamma)\Delta t\right]$$

If $\Delta t = 1$ timestep, then the proportion of individuals remaining in class C at the end of the timestep is $\exp[-(\alpha+\gamma)]$. The proportion of individuals leaving class C is $1-\exp[-(\alpha+\gamma)]$, of whom a fraction $\alpha/(\alpha+\gamma)$ go to class A and $\gamma/(\alpha+\gamma)$ go to class G.

To update the system we use the following convention. We calculate the total leaving rate for each model class, a vector called 'exit_rates'; for the above example the value of exit_rates would be $\alpha+\gamma$. In each timestep, beginning with a state vector X_{init}, we calculate the number of individuals that remain in class i as:

$Z[i]=\exp(-\text{exit\_rates}[i])X_{init}[i]$.

The number leaving class i is obviously $\{1-\exp(-\text{exit\_rates}[i])\}$ $X_{init}[i]$, but for compactness of notation we introduce an intermediate vector Y with elements:

$Y[i]= (1/\text{exit\_rates}[i])\{1-\exp(-\text{exit\_rates}[i])\}X_{init}[i]$.

Now if the per capita transition rate from class i to class j is α_{ij}, the number of individuals making the transition from i to j is simply $\alpha_{ij}Y[i]$.

In the above example, $\text{exit\_rates}[C]=\alpha+\gamma$, so $Z[C]=\exp[-(\alpha+\gamma)]$ $X_{init}[C]$ and $Y[C]=\{1/(\alpha+\gamma)\}\{1-\exp[-(\alpha+\gamma)]\}X_{init}[C]$. The transition rate from class C to class A is α, so the number of individuals making that transition in one timestep is $\alpha Y[C]=\{\alpha/(\alpha+\gamma)\}\{1-\exp[-(\alpha+\gamma)]\}X_{init}[C]$.

Core TB model equations

We first describe the model of TB infection and treatment, in the absence of MDR TB strains or HIV. The state vector X represents the number of individuals in each of the 42 TB states described in Table S1. Parameters used in the model are defined in Table S2. The total population is

$$N=\sum_{i=1}^{42}X[i]$$

The force of infection λ, describing the hazard rate of infection for each susceptible individual, is calculated from the weighted sum of all infectious classes:

$$\lambda=\frac{1}{N}\left\{\beta_P\left(X[4]+X[7]\right)+\beta_N\left(X[5]+X[8]\right)+\beta_{PD}\sum_{i=9}^{16}X[i]+\right.$$
$$\left.\beta_{PN}\sum_{i=17}^{24}X[i]+\beta_{ND}\sum_{i=26}^{33}X[i]+\beta_{NN}\sum_{i=34}^{41}X[i]+\beta_{PF}X[25]+\beta_{NF}X[42]\right\}$$

We now apply the competing rates formulation described above. The total exit rates for each state are described in Table S3.

We label the state vector at the beginning of a timestep X_{init}. The number of individuals remaining in each class is then given by

TABLE S2. Model parameters.

Greek symbols represent rates, lower case Roman symbols represent probabilities or proportions.

Symbol	Definition	Estimate (Range)
τ	Per capita birth rate (per 1000 population per year)	30
μ	Background mortality rate (per person per year):<br>*HIV-negative*<br>*HIV-positive*	 0.01<br>0.15
λ	Overall force of infection	computed
β_P	Number of new infections per untreated smear-positive case (per year) [a]	9.8 (7.0–12.6)
β_N	Number of new infections per untreated smear-negative case (per year) [b]	$0.15 \times \beta_P$
β_{PD}, β_{ND}, β_{NF}	Number of new infections per smear-positive or smear-negative case treated in a DOTS program, or per smear-negative case that has failed treatment (per year)	0
β_{PN}, β_{NN}	Number of new infections per smear-positive or smear-negative case treated in a non-DOTS programme (per year)	$0.25 \times \beta_P$<br>or $0.25 \times \beta_N$
β_{PF}	Number of new infections per smear-positive case that has failed treatment (per year) [a]	$0.5 \times \beta_P$<br>(0.25–0.75)
b	Relative susceptibility to reinfection for latent slow progressors or recovered cases [a]:<br>*HIV-negative*<br>*HIV-positive*	 0.35 (0.1–0.6)<br>0.75 (0.5–1.0)
f	Infectiousness (fitness) of MDR TB relative to drug-susceptible TB	0.40 (0.1–0.7)
p_S	Proportion of new TB infections entering latent slow-progressor pool [a]<br>*HIV-negative*<br>*HIV-positive*	 0.86 (0.75–0.92)<br>0.33 (0.20–0.64)
ϕ_S	Breakdown rate for slow progressors (endogenous reactivation) (per person per year) [a]<br>*HIV-negative*<br><br>*HIV-positive*	 0.000113<br>(0.0001–0.0003)<br>0.17 (0.04–0.20)
ϕ_F	Breakdown rate for fast progressors (per person per year) [a]	0.88 (0.76–0.99)
s_P	Proportion of new active cases with smear-positive disease<br>*HIV-negative* [b]<br>*HIV-positive* [a]	 0.45 (0.4–0.5)<br>0.3 (0.19–0.4)
σ	Rate of conversion from smear-negative to smear-positive disease (per person per year) [a]	0.015 (0.007–0.02)
π	Rate of spontaneous recovery from active to latent (per person per year) [a]	0.2 (0.15–0.25)
μ_P	TB fatality rate, untreated smear-positive (per person per year) [a]<br>*HIV-negative*<br>*HIV-positive*	 0.3 (0.2–0.4)<br>1.0 (0.5–1.0)
μ_N	TB fatality rate, untreated smear-negative (per person per year) [a]	$0.67 \times \mu_P$
d_P	Proportion of new active smear-positive cases eligible for detection [c]	see Figure S2
d_N	Proportion of new active smear-negative cases eligible for detection [a]	$0.6 \times d_P$
θ	Case detection rate (per person per year) [a,d]	4.0
t_P	Proportion of treated smear-positive cases under DOTS programs [c]	see Figure S2
t_N	Proportion of treated smear-negative cases under DOTS programs	0.4
δ_D	Default rate from DOTS treatment (per person per month) [d,e]	0.015
δ_N	Default rate from non-DOTS treatment (per person per month) [d,e]	$5 \times \delta_D$
v_D	Proportion of patients who complete but fail treatment under DOTS [d,e]	0.031
v_N	Proportion of patients who complete but fail treatment under non-DOTS [e]	$2 \times v_D$
ρ	Relapse rate from failed treatment classes to active disease (per person per year) [a]	0.3 (0.0–0.5)
a	Probability of developing MDR TB upon treatment failure [d,e]	0.03 (0.01–0.05)
μ_{PD}	TB mortality rate, smear-positive cases on DOTS treatment (per person per year) [d,e,f]	0.084
μ_{PN}	TB mortality rate, smear-positive cases on non-DOTS treatment (per person per year) [d,e,f]	0.1
μ_{ND}, μ_{NN}	TB mortality rate, smear-negative cases on DOTS or non-DOTS treatment (per person per year) [f]	$0.67 \times \mu_{PD}$<br>or $0.67 \times \mu_{PN}$

[a] Parameter values and ranges adapted from Dye and Williams [11] and Dye et al. [1].
[b] Adapted from Murray and Salomon [3].
[c] Derived from [12].
[d] Ranges in sensitivity analysis were defined as ±33% of the baseline values.
[e] Baseline values for treatment parameters were selected to match the estimates of 85% cure in DOTS programs and 50% cure in non-DOTS programs reported by Dye et al. [1] and to approximate regional outcomes reported in [12].
[f] Fatality rates for treated TB are assumed to be twice as high for HIV-positive patients.

the vector Z with elements:

$$Z[i] = \exp(-\text{exit\_rates}[i])X_{\text{init}}[i].$$

The intermediate vector Y has elements:

$$Y[i] = (1/\text{exit\_rates}[i])\{1-\exp(-\text{exit\_rates}[i])\}X_{\text{init}}[i]$$

We use these to calculate the state vector for the next timestep, X_{next} as listed in Table S4.

Note that in the equations referring to treatment states in the model, we include the "switch" variables K_D and K_N, which are vectors containing the monthly probabilities that a course of treatment ends. For conventional drug therapies, these are:

K_D=[0 0 0 0 0 1 0 0] for six-month DOTS treatment, and

K_N=[0 0 0 0 0 0 0 1] for eight-month non-DOTS treatment.

For the two-month treatment achieved with novel drug technologies, they are:

K_D=K_N= [0 1 0 0 0 0 0 0].

We assume, in this formulation, that shorter regimens, if available, would be used in both DOTS and non-DOTS treatment programs.

TABLE S3. Exit rates from states in the model.

State	Exit rates
1	$\lambda + \mu$
2	$\lambda b(1-p_S) + \phi_S + \mu$
3	$\phi_F + \mu$
4	$\pi + \mu_P + \mu$
5	$\pi + \sigma + \mu_N + \mu$
6	$\lambda b + \mu$
7	$\theta + \pi + \mu_P + \mu$
8	$\theta + \pi + \mu_N + \mu$
9–16	$\delta_D + \mu_{PD} + \mu$
17–24	$\delta_N + \mu_{PN} + \mu$
25	$\rho + \mu$
26–33	$\delta_D + \mu_{ND} + \mu$
34–41	$\delta_N + \mu_{NN} + \mu$
42	$\rho + \mu$

General analyses of treatment duration and tuberculosis dynamics

The core model described here was used first to consider the general relationships between treatment duration and tuberculosis dynamics. For these preliminary analyses, we did not incorporate heterogeneity in treatment programs (which is allowable through the "DOTS" / "non-DOTS" distinction noted above), and we focused exclusively on treatment of active, smear-positive pulmonary cases. We modeled the introduction of treatment into a stable equilibrium, following the example of Dye and colleagues [1].

TABLE S4. Equations for updating state vector at each timestep.

$$X_{\text{next}}[1] = Z[1] + \tau N$$

$$X_{\text{next}}[2] = Z[2] + p_S(\lambda Y[1] + b\lambda Y[6]) + \pi(Y[4] + Y[7] + Y[5] + Y[8])$$

$$X_{\text{next}}[3] = Z[3] + (1-p_S)(\lambda Y[1] + b\lambda Y[2] + b\lambda Y[6])$$

$$X_{\text{next}}[4] = Z[4] + s_P(1-d_P)(\phi_S Y[2] + \phi_F Y[3]) + \sigma Y[5] + (1-d_P)\rho Y[25]$$

$$X_{\text{next}}[5] = Z[5] + (1-s_P)(1-d_N)(\phi_S Y[2]+\phi_F Y[3]) + (1-d_N)\rho Y[42]$$

$$X_{\text{next}}[6] = Z[6] + \sum_{m=1}^{8}\{(1-v_D)K_D(Z[8+m]+Z[25+m]) + (1-v_N)K_N(Z[16+m]+Z[33+m])\}$$

$$X_{\text{next}}[7] = Z[7] + s_P d_P(\phi_S Y[2] + \phi_F Y[3]) + \sigma Y[8] + d_P\rho Y[25]$$

$$X_{\text{next}}[8] = Z[8] + (1-s_P)d_N(\phi_S Y[2] + \phi_F Y[3]) + d_N\rho Y[42]$$

$$X_{\text{next}}[9] = \theta t_P Y(7)$$

$$X_{\text{next}}[9+m] = Z[9+m-1](1-K_D[m]), \qquad m = 1,2,\ldots,7$$

$$X_{\text{next}}[17] = \theta(1-t_P)Y(7)$$

$$X_{\text{next}}[17+m] = Z[17+m-1](1-K_N[m]), \qquad m = 1,2,\ldots,7$$

$$X_{\text{next}}[25] = Z[25] + \sum_{m=1}^{8}\{\delta_D Y[8+m] + \delta_N Y[16+m] + v_D K_D[m]Z[8+m] + v_N K_N[m]Z[16+m]\}$$

$$X_{\text{next}}[26] = \theta t_N Y(8)$$

$$X_{\text{next}}[26+m] = Z[26+m-1](1-K_D[m]), \qquad m = 1,2,\ldots,7$$

$$X_{\text{next}}[34] = \theta(1-t_N)Y(8)$$

$$X_{\text{next}}[34+m] = Z[34+m-1](1-K_N[m]), \qquad m = 1,2,\ldots,7$$

$$X_{\text{next}}[42] = Z[42] + \sum_{m=1}^{8}\{\delta_D Y[25+m] + \delta_N Y[33+m] + v_D K_D[m]Z[25+m] + v_N K_N[m]Z[33+m]\}$$

Results from these preliminary analyses are reported in the main text.

To calculate the cumulative duration of infectiousness for a new treated smear-positive case, we accounted for the time before starting treatment (characterized by full infectiousness) and the time spent in the (partially infectious) failed state for those who default from treatment, with allowance for repeated cycles through active tuberculosis, treatment and possible failure for those who relapse from the failed state. We assumed for simplicity that individuals are not infectious while on treatment.

Starting with a single cycle of treatment, the duration of infectiousness is given by:

$$D + \mathrm{P(start)}\,\mathrm{P(fail)}\,T\,R$$

where D is the average delay from the onset of active disease to initiation of treatment; P(start) is the probability of initiating treatment, i.e. by avoiding pre-treatment mortality or remission; P (fail) is the probability of failing treatment for those who begin treatment (conditional on duration and default rate); T is the average residence time in the failed state following default; and R is the average relative infectiousness of individuals in the failed state.

A proportion of the individuals who fail treatment will eventually relapse, and a proportion of these relapsers will pass through a second cycle of treatment and possible failure; those failing a second cycle of treatment may relapse and begin a third cycle, and so on. The total duration of infectiousness allowing for these repeated cycles is described by the (infinite) series:

$$\begin{aligned} &D + \mathrm{P(start)}\,\mathrm{P(fail)}\,T\,R + \mathrm{P(fail)}\,\mathrm{P(relapse)}\,D \\ &\quad + \mathrm{P(fail)}\,\mathrm{P(relapse)}\,\mathrm{P(start)}\,\mathrm{P(fail)}\,T\,R \\ &\quad + \mathrm{P(fail)}\,\mathrm{P(relapse)}\,\mathrm{P(start)}\,\mathrm{P(fail)}\,\mathrm{P(relapse)}\,D \\ &\quad + \ldots \end{aligned}$$

where D, T, R, P(start) and P(fail) are as defined above, and P (relapse) indicates the proportion of failed cases who return to active tuberculosis.

Rewriting this equation as:

$$\begin{aligned} &[D + \mathrm{P(start)}\,\mathrm{P(fail)}\,T\,R] \\ &\quad \times [1 + \mathrm{P(fail)}\,\mathrm{P(relapse)} + \mathrm{P(fail)}^2\,\mathrm{P(relapse)}^2 + \ldots] \end{aligned}$$

we see that the second term in brackets is a power series, so we may simplify the expression to yield

$$[D + \mathrm{P(start)}\,\mathrm{P(fail)}\,T\,R] \,/\, [1 - \mathrm{P(fail)}\,\mathrm{P(relapse)}]$$

Results in the first panel of Figure 1 in the main text are based on this equation, with the value of D computed as $1 / (\mu_P + \mu + \pi + \theta)$; P(start) computed as $\theta / (\mu_P + \mu + \pi + \theta)$; T computed as $1 / (\mu + \rho)$; R set at 50% in the base case (with values of 25% and 75% considered in sensitivity analyses); P(fail) computed as:

$$\begin{aligned} &\sum_{j=1}^{m} \mathrm{P(default\ during\ month\ } j) \\ &\quad \times \mathrm{P(remain\ in\ treatment\ until\ the\ start\ of\ month\ } j) \end{aligned}$$

$$= \sum_{j=1}^{m} \delta / (\delta + \mu_T)(1 - \exp(-\delta - \mu_T))\exp(-(j-1)(\delta + \mu_T))$$

given treatment duration m, monthly default rate δ and monthly treatment fatality rate μ_T; and P(relapse) computed as $\rho / (\mu + \rho)$. For these calculations, we have taken $\mu_T = \mu + \mu_{PD}$ and defined parameter values for μ, μ_P, μ_{PD}, π, θ and ρ from the baseline values in Table S2. Note that we have assumed in these calculations that all treatment completion implies cure, i.e. that $\nu_D = 0$.

We estimated the effective reproductive number at time t from the model simulations of new infections and active infectious cases over time. The following approximation was used:

$$\begin{aligned} R_{eff} = &[(\mathrm{NewInf}_t)\,\mathrm{P(NewInf}\rightarrow\mathrm{Infectious)} \\ &+ (\mathrm{ReInf}_t)\,\mathrm{P(ReInf}\rightarrow\mathrm{Infectious)}] \\ &\times \mathrm{NewInfectious}_t / \mathrm{AllInfectious}_t \\ &\times \mathrm{Duration}_{avg} / \mathrm{NewInfectious}_t \end{aligned}$$

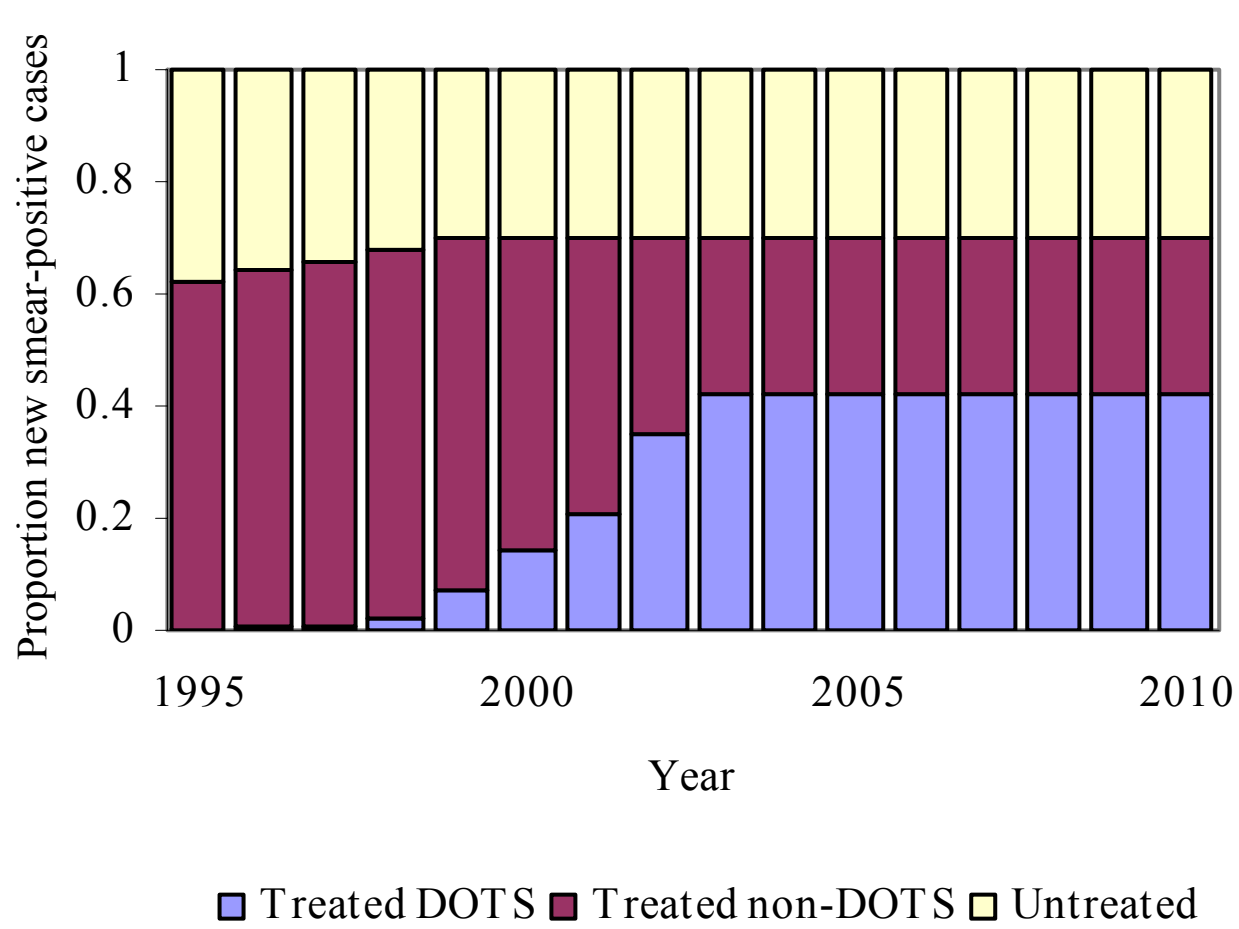

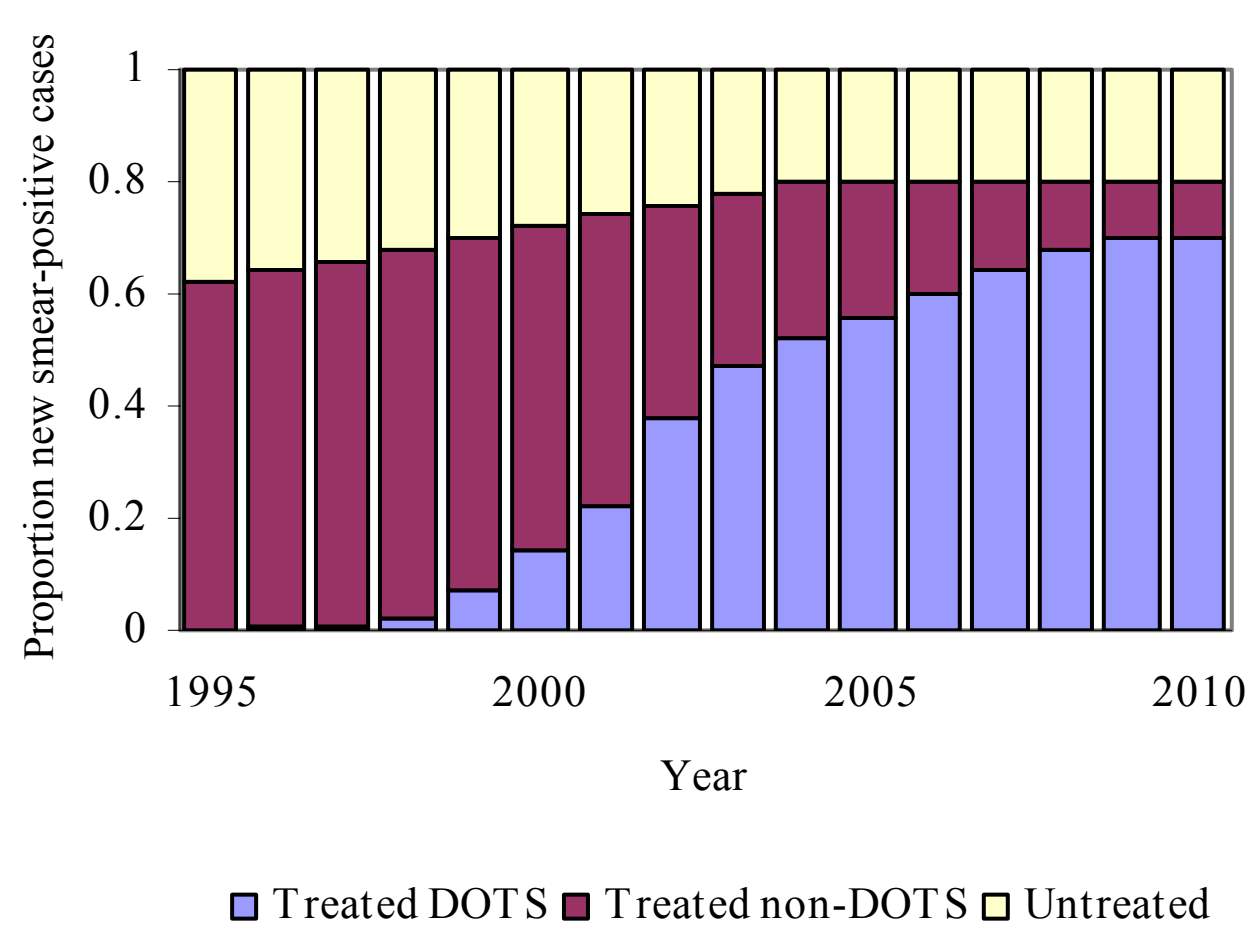

FIGURE S2. Case detection assumptions in two baseline scenarios.

Stable-DOTS scenario (left graph) assumes that detection of new smear-positive cases in DOTS areas remains stable at 42% starting in 2003. *DOTS-target* scenario (right graph) assumes that DOTS coverage reaches 70% by 2009.

= [(NewInf$_t$) P(NewInf→Infectious)
+ (ReInf$_t$) P(ReInf→Infectious)]
× Duration$_{avg}$ / AllInfectious$_t$

where NewInf$_t$ is the number of new infections at time t; ReInf$_t$ is the number of reinfections at time t, P(NewInf→Infectious) is the probability that a new infection will break down to active infectious tuberculosis (which includes all new smear-positive cases and a fraction β_N / β_P of new smear-negative cases); P (ReInf →Infectious) is the incremental probability that reinfection will lead to active infectious tuberculosis (i.e. reflecting only the additional breakdown probability for those reinfections that move from the slow to the fast breakdown category); NewInfectious$_t$ (which cancels out of the equation) is the incidence of new active infectious cases at time t, including new smear-positive cases and a fraction β_N / β_P of new smear-negative cases; Duration$_{avg}$ is the mean duration of new infectious cases, computed as a weighted average of the duration of a new smear-positive case (derived above) and the duration of an untreated smear-positive case, multiplied by the relative infectiousness of a smear-positive case β_N / β_P; and AllInfectious$_t$ is the prevalence, at time t, of all active infectious tuberculosis cases, including all untreated smear-positive cases, a fraction β_N / β_P of new smear-negative cases, and a fraction R (as defined above) of smear-positive cases who have failed treatment.

The estimate of average change in incidence over time follows the example of Dye and colleagues [1], derived by first computing the change in incidence rates from year to year and then averaging these annual changes over 20 years following the introduction of treatment.

Finally, we computed the direct and indirect mortality benefits of treatment as follows. First, the direct mortality reductions were estimated by calculating the probability of dying for a new active tuberculosis case with and without treatment. For untreated tuberculosis the probability of dying, computed separately for smear-positive and smear-negative patients, is μ_U / (μ_U +π), where μ_U combines background mortality with the type-specific untreated fatality rate, and π is the spontaneous cure rate. A weighted average of the smear-positive and smear-negative mortality probabilities was taken, with weights reflecting the composition of new active cases.

For treated smear-positive tuberculosis, in similar fashion to the calculation of the cumulative duration of infectiousness described above, we allowed for repeated cycles of treatment and failure, so that the total probability of death for a treated case is described by:

P(pre-Tx death)+P(start) P(Tx death)
+ P(start) P(fail) P(fail death)
+ P(start) P(fail) P(relapse)
× [P(pre-Tx death) + P(start) P(Tx death)]
+ P(start) P(fail) P(relapse) P(start) P(fail) P(fail death)
+ …

where P(fail) and P(relapse) are defined as above; P(pre-Tx death) is the probability of dying before initiating treatment; P(Tx death) is the probability of dying while on treatment; and P(fail death) is the probability of dying while residing in the failed state. This expression simplifies to

[P(pre-Tx death) + P(start) P(Tx death)
+ P(start) P(fail) P(fail death)]
× [1 + P(start) P(fail) P(relapse)
+ P(start)^2 P(fail)^2 P(relapse)^2 + …]

which is a power series that simplifies further to

[P(pre-Tx death) + P(start) P(Tx death)
+ P(start) P(fail) P(fail death)] /
[1 – P(start) P(fail) P(relapse)]

In this expression, P(start), P(fail) and P(relapse) are computed as above; P(pre-Tx death) is computed as (μ_P + μ) / (μ_P + μ + π + θ); P(Tx death) is computed analogously to P(fail) as a function of the default rate and treatment duration:

$$\sum_{j=1}^{m} \mu_T / (\delta + \mu_T)(1 - \exp(-\delta - \mu_T))\exp(-(j-1)(\delta + \mu_T))$$

and P(fail death) is computed as μ / (μ +ρ).

The total reduction in cumulative mortality, compared to a steady-state, *no treatment* counterfactual, was estimated directly from simulations over a 20-year period. The component of that reduction that is due to direct benefits of introducing treatment (reported in Figure 2 in the main text), will be approximately equal to the per-patient reduction in the treatment fatality risk.

Extensions to the core model: multi-drug resistant TB and TB-HIV

The core model was extended to allow for the development of MDR strains through poor treatment and subsequent transmission of resistant strains. We replicated all 42 categories in the core TB model to allow for parallel MDR and non-MDR states. Develop-

TABLE S5. Partial rank correlation coefficients for key variables with respect to reductions in incidence in 2030 relative to *stable-DOTS* baseline; and reductions in incidence in 2030 relative to *DOTS-target* baseline.

Parameter or variable	Stable-DOTS	DOTS-target
Proportion of new TB infections entering latent slow-progressor pool (HIV-negative)	–0.73	–0.86
Number of new infections per SS+ case that has failed treatment (per year)	0.60	0.63
Number of new infections per untreated SS+ case (per year)	0.31	0.55
Monthly default probability (DOTS)	0.50	0.52
Relapse rate from failed treatment class to active disease	–0.19	–0.56
Endogenous reactivation rate (HIV-negative)	–0.19	–0.15
Relative susceptibility to reinfection for latent slow progressors or recovered	0.15	0.25
Monthly default probability (non-DOTS)	0.27	0.09
Endogenous reactivation rate (HIV-positive)	–0.15	–0.15

ment of MDR may occur among those who default from treatment or those who complete but fail treatment, at a specified probability (*a*). Transmission of MDR strains may differ from transmission of non-MDR strains, which we model by multiplying the transmission rate by a 'fitness' parameter *f*.

We also accounted for the interaction between TB and HIV-1 by replicating all (42×2) model states again to allow for two different categories of HIV status: uninfected and infected. Several key natural history parameters vary according to HIV state, including mortality rates, probabilities of developing primary progressive TB within the first few years after infection, and long-term breakdown rates. HIV incidence was incorporated as an exogenous input to the model, which varies over time to capture relevant trends. Regional estimates of HIV incidence trends were developed by WHO and UNAIDS based on methods described elsewhere [5,6]. We assumed a three-year period between HIV infection and increased rates of breakdown to active tuberculosis. Previous models have assumed a somewhat longer delay [7], but a recent report indicates that the rise in risk may in fact occur even sooner than three years after HIV infection [8]. We examined alternative values of this delay from 1 year to 5 years in univariate sensitivity analyses and found that the results reported here were robust to variation in this assumption.

Initial conditions and regional calibration

To calibrate regional models to present conditions in order to examine the potential future impact of new drugs, we ran a series of simulations with parameters representing different phases in TB history. First, a virgin epidemic was simulated, in which one infectious source case is introduced in a population of susceptibles. This epidemic was run to equilibrium, which was assumed to represent mid-century conditions in many developing regions, prior to the introduction of chemotherapy. We then introduced treatment into the model in stages, reflecting a period of sub-optimal treatment and, eventually, expanding coverage of DOTS.

Parameters relating to treatment coverage and outcomes were drawn from WHO reports, and calibration to regional epidemiologic data was undertaken in reference to WHO estimates of incidence, prevalence, mortality, and MDR prevalence among new cases for the year 2002.

Sensitivity and uncertainty analysis

Following the approach developed by Iman et al. [9,10] and implemented by Dye and colleagues in their previous analysis of tuberculosis control strategies [1], we undertook sensitivity and uncertainty analyses for the regional results based on multiple simulations of the model with varying parameter values. First, 1000 values were sampled from independent triangular distributions using latin hypercube sampling, with the peak values and upper and lower limits defined in Table S2, or upper and lower limits defined as ±33% of baseline values for program variables (case detection, default, failure and treatment fatality rates). The model was recomputed for each set of sampled parameter values, including all stages of the analysis, from the virgin epidemic through the evolving treatment programs. In order to assess the importance of different model parameters we computed partial rank correlation coefficients of various input parameters in relation to the reductions in incidence and mortality in 2015 and 2030 under different scenarios compared to the stable DOTS and DOTS-target baselines. Ranges around key outputs from the model reported in the main text were based on the 2.5^{th} and 97.5^{th} centiles across the 1000 simulations.

Results from the sensitivity analysis are shown in Table S5. Partial rank correlation coefficients are reported for key variables in relation to two different outcome variables: percent reduction in annual incidence by 2030 under a shortened regimen compared to (a) the *stable DOTS* baseline, and (b) the *DOTS target* baseline. Estimated benefits of shortening drug regimens rise with increasing default rates and infectiousness—particularly infectiousness of treatment failures. Estimated benefits fall when endogenous reactivation rates are high or the proportion of new infections that do not progress quickly to active disease rises, since these changes give relatively greater weight to past trends in infection (which are less amenable to change through treatment) as determinants of incidence. Estimated benefits also fall when the relapse rate from the failed treatment class increases—since residence in the treatment failure class is marked by lower mortality risks than active TB combined with partial infectiousness, shorter durations in failed treatment will diminish the negative impact of poor treatment.

References

1. Dye C, Garnett GP, Sleeman K, Williams BG (1998) Prospects for worldwide tuberculosis control under the WHO DOTS strategy. Directly observed short-course therapy. Lancet 352: 1886-1891.
2. Blower SM, Small PM, Hopewell PC (1996) Control strategies for tuberculosis epidemics: new models for old problems. Science 273: 497-500.
3. Murray CJ, Salomon JA (1998) Modeling the impact of global tuberculosis control strategies. Proc Natl Acad Sci U S A 95: 13881-13886.
4. Cohen T, Murray M (2004) Modeling epidemics of multidrug-resistant M. tuberculosis of heterogeneous fitness. Nat Med 10: 1117-1121.
5. Ghys PD, Brown T, Grassly NC, Garnett G, Stanecki KA, Stover J, Walker N (2004) The UNAIDS Estimation and Projection Package: a software package to estimate and project national HIV epidemics. Sex Transm Infect 80 Suppl 1: i5-i9.
6. Walker N, Stover J, Stanecki K, Zaniewski AE, Grassly NC, Garcia-Calleja JM, Ghys PD (2004) The workbook approach to making estimates and projecting future scenarios of HIV/AIDS in countries with low level and concentrated epidemics. Sex Transm Infect 80 Suppl 1: i10-i13.
7. Currie CS, Williams BG, Cheng RC, Dye C (2003) Tuberculosis epidemics driven by HIV: is prevention better than cure? AIDS 17: 2501-2508.
8. Sonnenberg P, Glynn JR, Fielding K, Murray J, Godfrey-Faussett P, Shearer S (2005) How soon after infection with HIV does the risk of tuberculosis start to increase? A retrospective cohort study in South African gold miners. J Infect Dis 191: 150-158.
9. Iman RL, Helton JC, Campbell JE (1981) An approach to sensitivity analysis of computer models: part I—introduction, input variable selection and preliminary variable assessment. Journal of Quality Technology 13: 174-183.
10. Iman RL, Helton JC, Campbell JE (1981) An approach to sensitivity analysis of computer models: part II—ranking of input variables, response surface validation, distribution effect, and technique synopsis variable assessment. Journal of Quality Technology 13: 232-240.
11. Dye C, Williams BG (2000) Criteria for the control of drug-resistant tuberculosis. Proc Natl Acad Sci U S A 97: 8180-8185.
12. World Health Organization (2005) WHO report 2005: global tuberculosis control: surveillance, planning and financing (WHO/HTM/TB/2005.349).
