# Supplementary material for: Prospects for Advancing Tuberculosis Control Efforts through Novel Therapies
Source: PLoS Med. 2006 Aug 1;3(8):e273. doi: 10.1371/journal.pmed.0030273 (PMC1523376; doi:10.1371/journal.pmed.0030273)
Supplement: Supplementary file 2 [file pmed.0030273.sg001.pdf]

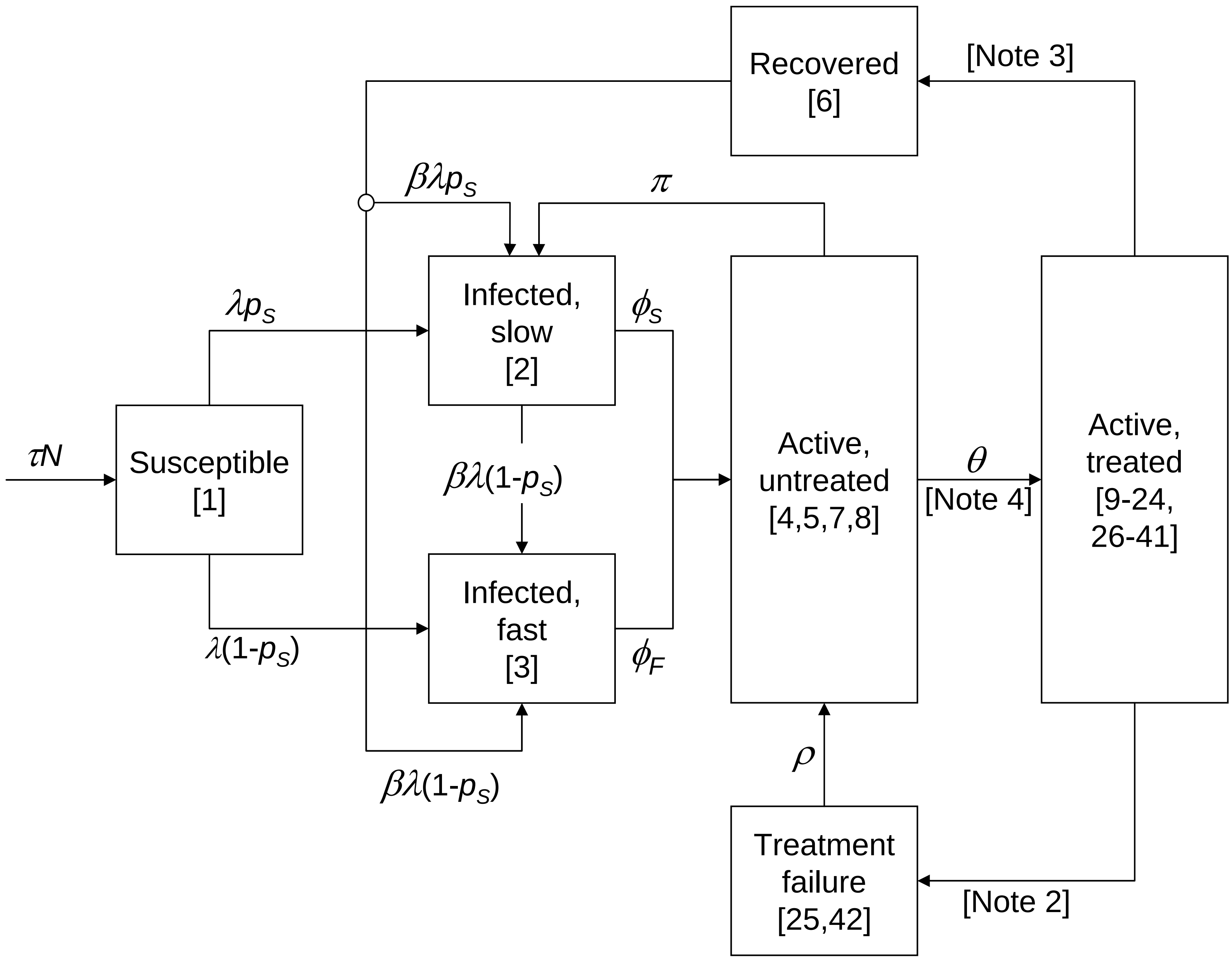
Recovered
[6]
[Note 3]
$\beta\lambda p_S$
π
λp_S
Infected,
slow
[2]
ϕ_S
τN
Susceptible
[1]
$\beta\lambda(1-p_S)$
Active,
untreated
[4,5,7,8]
θ
[Note 4]
Active,
treated
[9-24,
26-41]
Infected,
fast
[3]
$\lambda(1-p_S)$
ϕ_F
$\beta\lambda(1-p_S)$
ρ
Treatment
failure
[25,42]
[Note 2]
