# Supplementary material for: Prospects for Advancing Tuberculosis Control Efforts through Novel Therapies
Source: PLoS Med. 2006 Aug 1;3(8):e273. doi: 10.1371/journal.pmed.0030273 (PMC1523376; doi:10.1371/journal.pmed.0030273)
Supplement: Supplementary file 3 [file pmed.0030273.sg002.pdf]

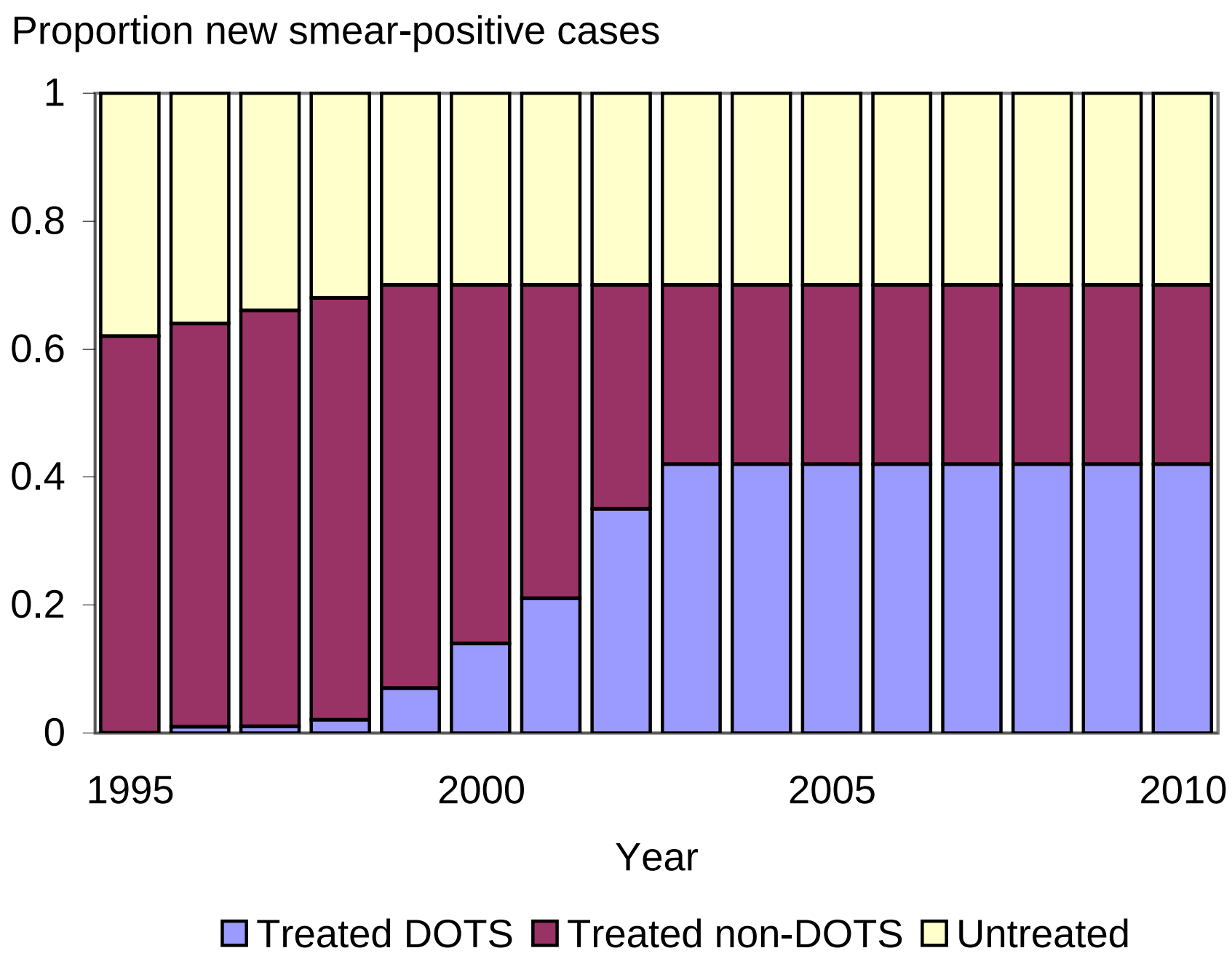

Proportion new smear-positive cases
1
0.8
0.6
0.4
0.2
0
1995
2000
2005
2010
Year
Treated DOTS
Treated non-DOTS
Untreated

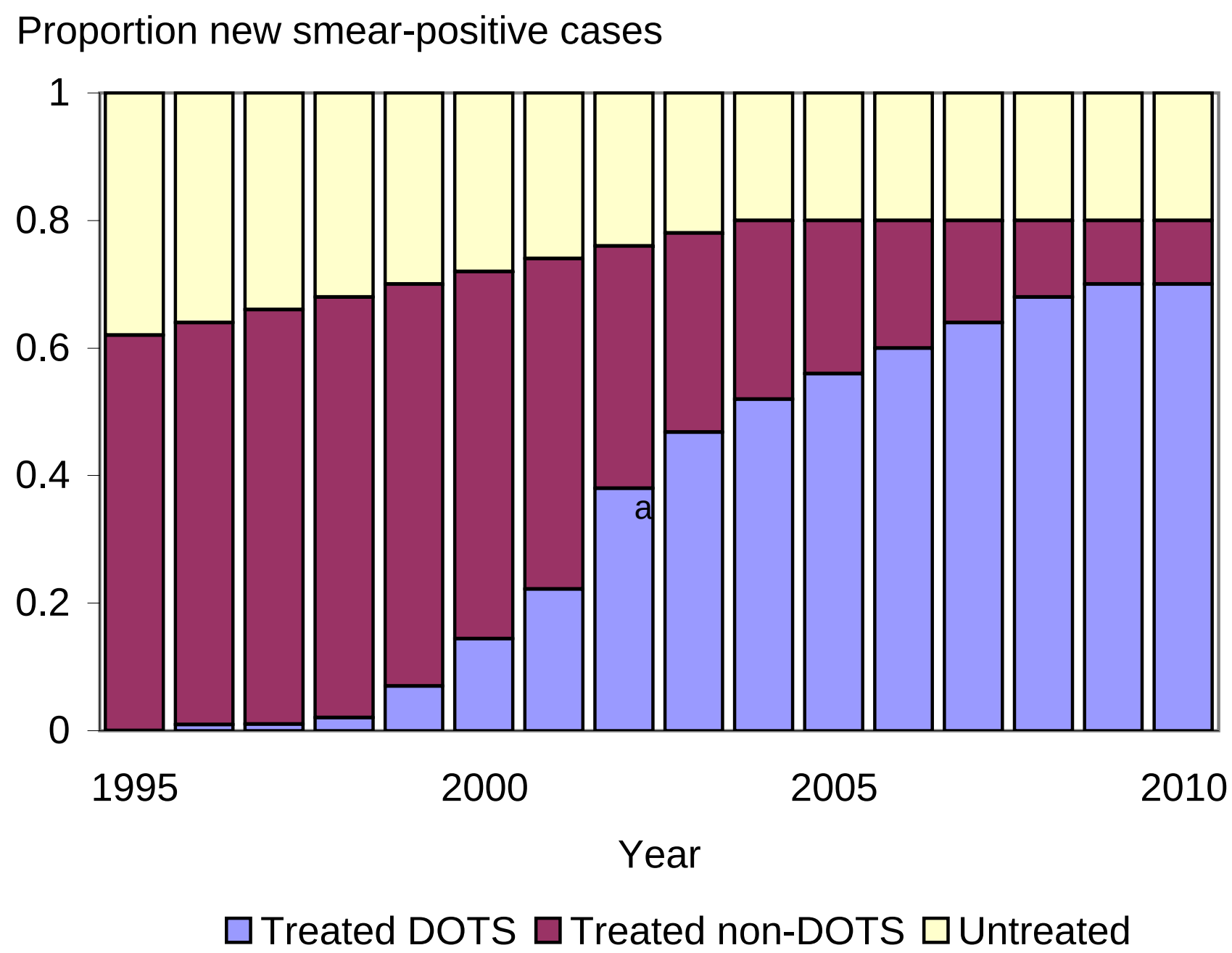

Proportion new smear-positive cases
1
0.8
0.6
0.4
0.2
0
a
1995
2000
2005
2010
Year
Treated DOTS
Treated non-DOTS
Untreated

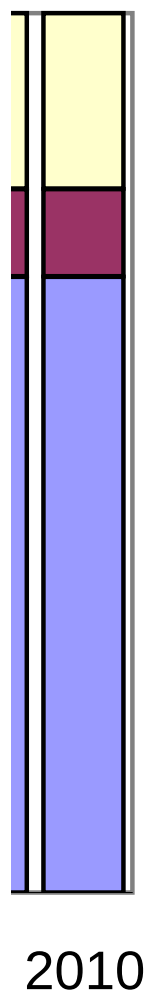
2010
