# Supplementary material for: Prospects for Advancing Tuberculosis Control Efforts through Novel Therapies
Source: PLoS Med. 2006 Aug 1;3(8):e273. doi: 10.1371/journal.pmed.0030273 (PMC1523376; doi:10.1371/journal.pmed.0030273)
Supplement: Supplementary file 4 [file pmed.0030273.sg003.pdf]

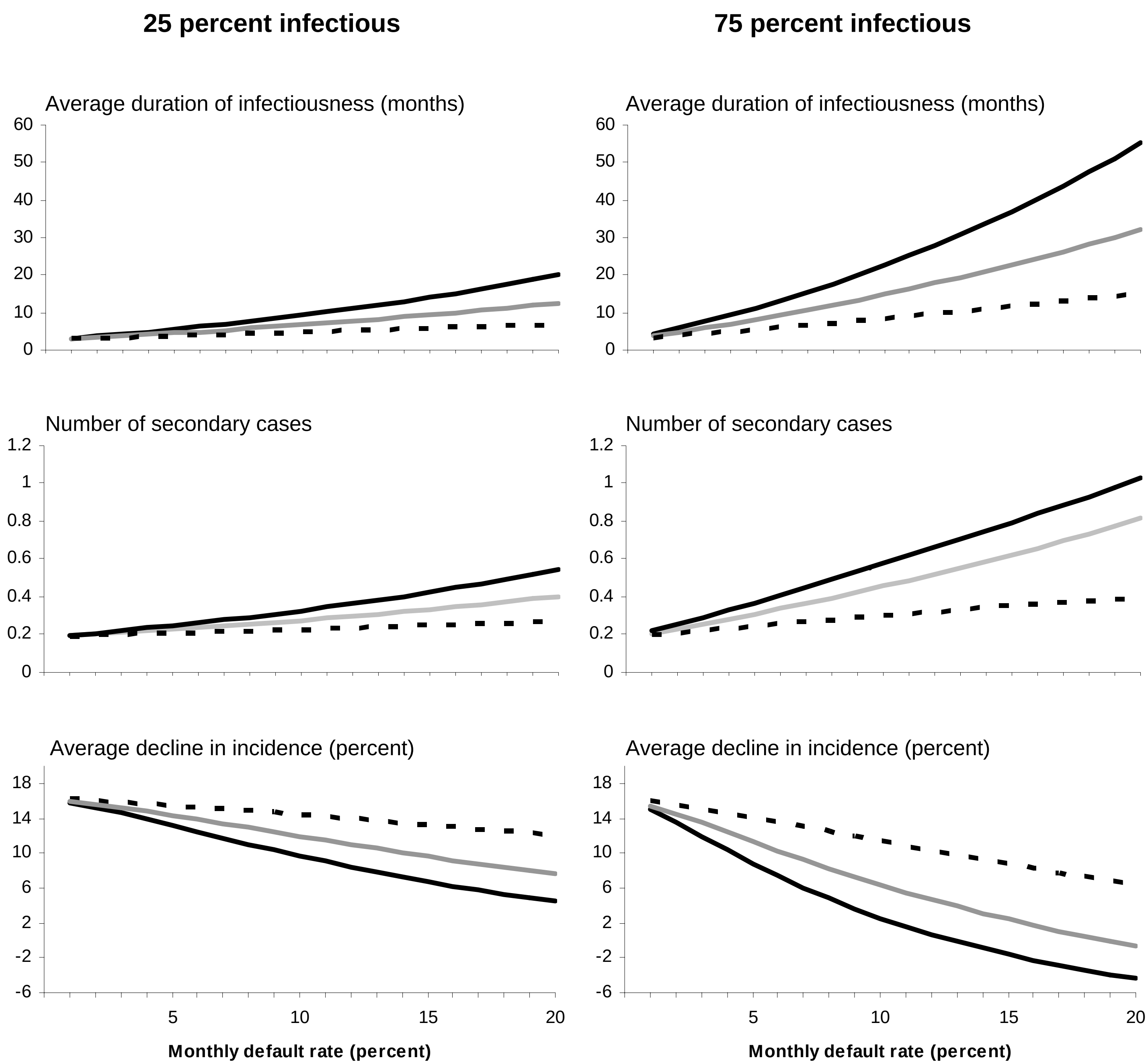
25 percent infectious
75 percent infectious
Average duration of infectiousness (months)
60
50
40
30
20
10
0
Average duration of infectiousness (months)
60
50
40
30
20
10
0
Number of secondary cases
1.2
1
0.8
0.6
0.4
0.2
0
Number of secondary cases
1.2
1
0.8
0.6
0.4
0.2
0
Average decline in incidence (percent)
18
14
10
6
2
-2
-6
5
10
15
20
Monthly default rate (percent)
Average decline in incidence (percent)
18
14
10
6
2
-2
-6
5
10
15
20
Monthly default rate (percent)
